# Tracking adverse drug reactions and medication errors in the Central Chronic Medicine Dispensing and Distribution (CCMDD) programme in South Africa

**DOI:** 10.4102/sajhivmed.v23i1.1366

**Published:** 2022-05-19

**Authors:** Kennedy Otwombe, Maggie Munsamy, Mukesh Dheda, Nishana Ramdas, Corlee Herbst, Merlin Pillay, Tanya van Tonder, Celicia Serenata, Samanta Lalla-Edward

**Affiliations:** 1 Perinatal HIV Research Unit, Faculty of Health Sciences, University of the Witwatersrand, Johannesburg, South Africa; 2 School of Public Health, Faculty of Health Sciences, University of the Witwatersrand, Johannesburg, South Africa; 3 Central Chronic Medicines Dispensing and Distribution (CCMDD) Programme, National Department of Health, Pretoria, South Africa; 4 Pharmacovigilance Programme for Public Health, National Department of Health, Pretoria, South Africa; 5 Opinion Solutions, Johannesburg, South Africa; 6 Ezintsha, Faculty of Health Sciences, University of the Witwatersrand, Johannesburg, South Africa

**Keywords:** CCMDD, National Health Insurance, ADRs, Medication errors, HIV

## Abstract

**Background:**

The South African Central Chronic Medicine Dispensing and Distribution (CCMDD) programme is a National Health Insurance (NHI) initiative that improves access to medicine for patients.

**Objectives:**

To describe the frequency of adverse drug reactions (ADRs) and medication errors reported in stable patients living with HIV.

**Method:**

This descriptive cross-sectional survey was conducted from August 2020 to October 2020, targeting tenofovir disoproxil fumarate/lamivudine/dolutegravir (TLD) and tenofovir disoproxil fumarate/emtricitabine/efavirenz (TEE) patients. The distribution of ADRs and medication errors is presented.

**Results:**

Of 9621 patients, 30.8% (*n* = 2967) were interviewed, 40.2% (*n* = 1192) on TLD and 59.8% (*n* = 1775) on TEE regimens. The majority were women (TLD: 55.8%, *n* = 665; TEE: 75.4%, *n* = 1338); 15% (179/1192) reported ADRs on TLD. Medication errors were low on TLD (1.6%, *n* = 19) and TEE (1.2%, *n* = 22). Receipt of incorrect medication (eight each in TLD and TEE) and associated hospitalisations (one vs two, respectively) were low. Common TLD-associated ADRs were weight gain (47.5%, *n* = 85), headaches (44.7%, *n* = 80), insomnia (39.7%, *n* = 71), restlessness (36.9%, *n* = 66), dizziness (29.6%, *n* = 53), brain fog (27.9%, *n* = 50), nervousness (27.4%, *n* = 49), rash on the skin (24.6%, *n* = 44) and poor concentration (21.2%, *n* = 38).

**Conclusion:**

About one in seven patients reported ADRs under TLD. Medication errors were low, possibly due to effective quality control measures and stable patients being on the programme. Knowing the frequency of ADRs and medication errors is critical for enhancing the CCMDD programme.

## Introduction

The demand for affordable health services prompted the South African government to roll out the National Health Insurance (NHI) cover. In brief, the NHI seeks to ensure access to a defined package of comprehensive and essential healthcare services for all its citizens, irrespective of their financial status, and to achieve sustainable development goal ‘3’ of ensuring healthy lives and promoting the well-being of all South Africans.^1,2^ The NHI grant funds the Central Chronic Medicine Dispensing and Distribution (CCMDD) programme with the objective of improving patients’ access to chronic medicines through the public health system and enhancing their healthcare experiences.^3^ The CCMDD programme delivers pre-dispensed medication to contracted pick-up points offering a larger service footprint for patients which increases convenience and accessibility. This is achieved by harnessing public-private partnerships. Stable patients, defined as being adherent to their treatment and clinically well as per specific disease guidelines, are invited to participate in the programme and collect their medicine parcel every 2–3 months at their chosen pick-up point; these include pharmacies, doctors’ rooms, innovative hubs, smart locker systems and community centres. The patients are provided a 6-month repeat prescription and return to a health facility once every six months, or when they experience any health problems.

The CCMDD programme has been implemented in South Africa since February 2014, when it was first initiated as a pilot programme in 11 NHI pilot districts. Subsequently, the programme significantly expanded to eight provinces (excluding the Western Cape).^4^ According to the most recent report by the Joint United Nations Programme on HIV/AIDS, South Africa has the largest number of people living with HIV (PLWH) and on antiretroviral treatment (ART).^5^ Many of these people receive their ART through the CCMDD programme.^6^ There are more than 4 million patients registered on the programme; 75% are PLWH (of these, 65% were on ART only and 35% on ART and medication for co-morbidities), and 25% are HIV-uninfected and on treatment for non-communicable diseases.

Medication errors in the programme, defined as failure in the treatment process that lead to, or have the potential to cause, harm to the patient,^7,8^ are isolated. An adverse drug reaction (ADR) is any harmful and unwanted reaction to medication that occurs at doses normally prescribed to patients for therapeutic or prophylactic purposes.^9,10^ Few ADR cases are reported in the CCMDD programme. Patients on the CCMDD programme are counselled to report any ADRs or medication errors experienced. The CCMDD team receives reports of ADRs and medication errors (classified according to predefined categories of harm) and this is shared with the relevant provinces and districts as well as with the National Department of Health pharmacovigilance unit. Where the fault of the error falls on the service provider, the service provider accepts the responsibility of contacting the patient and, if needed, ensures that the patient receives medical attention. Patients can also self-report ADRs to the South African Health Products Regulatory Authority.

While data on ADRs from differentiated service delivery models similar to the CCMDD programme for PLWH are commonly reported in low- and middle-income countries, reporting of medication errors remains sparse.^11,12^ This survey describes the frequency of ADRs and medication errors reported by stable PLWH that receive their ART medication through the CCMDD programme. We present the most commonly reported ADRs under the tenofovir disoproxil fumarate/lamivudine/dolutegravir (TLD) regimen and medication errors reported on TLD and tenofovir disoproxil fumarate/emtricitabine/efavirenz (TEE) regimens.

## Research methods and design

### Study design

This was a descriptive cross-sectional survey conducted between 24 August 2020 and 12 October 2020 to track ADRs and medication errors reported by patients in the CCMDD programme. All responses to the occurrence of ADRs and medication errors were self-reported by those interviewed.

### Study setting

Stable patients in the CCMDD programme were identified from the operational database and contacted to participate in the survey. They were recruited from eight provinces, excluding the Western Cape (which runs an independent programme).

### Study population and sampling strategy

Clinicians from public healthcare facilities identify stable patients with chronic diseases, including HIV, for possible inclusion in the CCMDD programme. The programme is opt-in, allowing patients to choose to be included or not. Once an eligible patient agrees to participate in the CCMDD programme, a 6-month repeat prescription is completed. The patient is given a list of contracted pick-up points from which to choose to collect their medication parcel.

The study enrolled patients with chronic diseases, aged ≥ 18 years. Two key categories were selected for inclusion in our analysis as they comprised the largest proportion of patients in the CCMDD programme:

- Patients receiving the TLD regimen: to collect information on the frequency of ADRs and medication errors and to establish whether the correct patients were transitioned as per the CCMDD TLD standard operating procedure.
- Patients on the TEE regimen: to determine the frequency of ADRs and medication errors and to establish why TLD was not offered to them, or why patients chose to remain on TEE despite the availability of TLD.

Multi-stage sampling was used to select the patients who were interviewed. Patients from each province were identified and then proportionately selected for interviews for both TLD and TEE drug regimens. Within the categories, simple random sampling was used to select the patients to be interviewed.

### Sample size

The sample size was determined using the method for prevalence:


$$n=\left[z^{2}p(1-p)\right]/d^{2},$$ [Eqn 1]

where *n* = sample size, *z* = z-statistic for level of confidence, *p* = expected prevalence and *d* = precision. Approximately 70% of patients in the CCMDD programme are living with HIV and are almost evenly distributed between TLD and TEE regimens. Assuming about 35% of the PLWH in the CCMDD programme are on the TLD regimen, at least 1115 would be surveyed, with a precision of 2.8%. Similarly, at least 1653 would be surveyed, with a 2.3% precision, assuming an equivalent proportion on the TEE regimen.

### Data collection

Prior to data collection, a survey questionnaire was developed based on the aims and objectives of the study. Items of interest included: socio-demographic information; episodes of ADRs and medication errors; whether the ADRs occurred because of medication errors; how ADRs and medication errors were handled by the CCMDD programme; whether the medication errors were identified by the patients, health facility staff or service provider; and whether the correct medication was issued thereafter. The initial questionnaire was pre-tested on a small random sample of patients to assess its suitability. Responses from pre-testing were used to guide improvements to the final questionnaire. Some of the items in the questionnaire, such as weight gain, were open-ended to allow for diversity in responses, whereas others were categorised. The open-ended items were re-categorised following discussions with the study team, for purposes of statistical reporting.

Data collection was conducted using digital platforms (unstructured supplementary service data, Upinion Webapp and a mobi-site) and a call centre where staff were trained on the CCMDD programme. A text message was sent to all patients who had made a visit to CCMDD medication sites in the 6 months prior to the survey, excluding known deaths. They were informed that the CCMDD programme was collecting information for a health report. This was followed up with a multimedia messaging service that contained study information and a consent form. Only those who agreed to participate were invited to select their preferred mode of survey completion. If no response was received within three days of the last attempt, a trained call centre agent called the patient. An electronic data capturing system was used to capture the survey questionnaire responses in real time via all the digital platforms and the call centre. The call centres operated for extended hours on weekdays, Saturdays and public holidays. Data were reviewed after each interview and queries were addressed in real time. The database was de-identified and all personal identifiers were redacted prior to conducting statistical analysis.

### Data analysis

Frequencies and proportions were determined for categorical data that were stratified by province. Statistical analysis was conducted using SAS Enterprise Guide 7.15 (2017, SAS Institute, Cary, NC, United States).

### Ethical considerations

Ethical approval to conduct this study was provided by the University of the Witwatersrand Human Research Ethics Committee (ethics clearance number M201195). Survey respondents did not receive any incentives.

## Results

Of the initial sample size of 9621, a total of 2967 (30.8%) patients, stable on their HIV treatment, were interviewed.

### Tenofovir disoproxil fumarate/lamivudine/dolutegravir regimen

Table 1 presents participant characteristics of patients on the TLD regimen, stratified by province. A total of 1192 patients on the TLD regimen were interviewed. Overall, the majority were women (55.8%, *n* = 665) and the age groups 40–49 (36.3%, *n* = 433), 30–39 (26%, *n* = 310) and 50–59 (24.5%, *n* = 292) years had the highest proportion of responses. The majority had been on the dolutegravir (DTG)-containing regimen, TLD (37.3%, *n* = 445), for at least 6 months. Among females of childbearing age (20–49 years), 55.4% (*n* = 246) reported being counselled regarding the risk of DTG and pregnancy.

**TABLE 1:** Participant characteristics of patients on the tenofovir disoproxil fumarate/lamivudine/dolutegravir regimen.

Variables	Overall		Gauteng		KwaZulu-Natal		Eastern Cape		Free State		Limpopo		Mpumalanga		North West	
	*n*	%	*n*	%	*n*	%	*n*	%	*n*	%	*n*	%	*n*	%	*n*	%
Number interviewed (*N*)	1192	-	402	-	323	-	156	-	106	-	28	-	64	-	113	-
**Gender**																
Female	665	55.8	222	55.2	155	48.0	102	65.4	67	63.2	15	53.6	34	53.1	70	61.9
Male	527	44.2	180	44.8	168	52.0	54	34.6	39	36.8	13	46.4	30	46.9	43	38.1
**Age Group (years)**																
20–29	53	4.4	10	2.5	11	3.4	15	9.6	7	6.6	1	3.6	1	1.6	8	7.1
30–39	310	26.0	100	24.9	99	30.7	38	24.4	28	26.4	2	7.1	15	23.4	28	24.8
40–49	433	36.3	156	38.8	107	33.1	53	34.0	38	35.8	9	32.1	27	42.2	43	38.1
50–59	292	24.5	99	24.6	84	26.0	34	21.8	26	24.5	10	35.7	19	29.7	20	17.7
60–69	81	6.8	29	7.2	15	4.6	13	8.3	5	4.7	5	17.9	1	1.6	13	11.5
70–79	17	1.4	7	1.7	4	1.2	3	1.9	2	1.9	0	0.0	0	0.0	1	0.9
Missing	6	0.5	1	0.2	3	0.9	0	0.0	0	0.0	1	3.6	1	1.6	0	0.0
**How long (in months) have you been taking the new DTG-containing regimen?**																
1 month	130	10.9	43	10.7	27	8.4	21	13.5	12	11.3	7	25.0	8	12.5	12	10.6
2 months	217	18.2	80	19.9	50	15.5	31	19.9	18	17.0	2	7.1	11	17.2	25	22.1
3 months	198	16.6	70	17.4	56	17.3	22	14.1	16	15.1	8	28.6	13	20.3	13	11.5
4 months	113	9.5	37	9.2	33	10.2	12	7.7	11	10.4	3	10.7	6	9.4	11	9.7
5 months	47	3.9	17	4.2	14	4.3	8	5.1	4	3.8	0	0.0	1	1.6	3	2.7
6 months or longer	445	37.3	130	32.3	143	44.3	54	34.6	42	39.6	8	28.6	25	39.1	43	38.1
Missing	42	3.5	25	6.2	0	0.0	8	5.1	3	2.8	0	0.0	0	0.0	6	5.3
**If female: Have you been counselled on the risk of DTG and pregnancy?** †																
Missing	33	7.4	17	11.7	1	1.0	7	9.9	2	4.3	1	16.7	0	0.0	5	10.0
No	165	37.2	49	33.8	44	42.3	25	35.2	19	41.3	3	50.0	11	50.0	14	28.0
Yes	246	55.4	79	54.5	59	56.7	39	54.9	25	54.3	2	33.3	11	50.0	31	62.0
**Are you taking any other medication together with your DT G regimen?**																
Missing	44	3.7	25	6.2	2	0.6	8	5.1	3	2.8	0	0.0	0	0.0	6	5.3
No	978	82.0	294	73.1	298	92.3	132	84.6	79	74.5	26	92.9	61	95.3	88	77.9
Yes	170	14.3	83	20.6	23	7.1	16	10.3	24	22.6	2	7.1	3	4.7	19	16.8

DTG, Dolutegravir.

† , Refers to females of childbearing age in this sample aged between 20 and 49 years.

Table 2 presents overall ADRs reported by respondents by province. Adverse drug reactions on the TLD regimen were reported by 15% (*n* = 179) of the respondents. Limpopo (28.6%, *n* = 8), KwaZulu-Natal (23.2%, *n* = 75) and Mpumalanga (21.9%, *n* = 14) provinces had the highest proportion of ADRs. The most commonly reported ADRs were weight gain (47.5%, *n* = 85), headaches (44.7%, *n* = 80), insomnia (39.7%, *n* = 71), restlessness (36.9%, *n* = 66), dizziness (29.6%, *n* = 53), brain fog (27.9%, *n* = 50), nervousness (27.4%, *n* = 49), skin rash (24.6%, *n* = 44) and poor concentration (21.2%, *n* = 38).

**TABLE 2:** Adverse drug reactions reported by patients on the tenofovir disoproxil fumarate/lamivudine/dolutegravir regimen in the survey.

Variables	Overall		Gauteng		KwaZulu-Natal		Eastern Cape		Free State		Limpopo		Mpumalanga		North West	
	*n*	%	*n*	%	*n*	%	*n*	%	*n*	%	*n*	%	*n*	%	*n*	%
**Have you experienced any side effects when taking the DTG treatment?**																
Missing	109	9.1	67	16.7	1	0.3	19	12.2	9	8.5	0	0.0	0	0.0	13	11.5
No	904	75.8	296	73.6	247	76.5	119	76.3	83	78.3	20	71.4	50	78.1	89	78.8
Yes	179	15.0	39	9.7	75	23.2	18	11.5	14	13.2	8	28.6	14	21.9	11	9.7
**Have you experienced weight gain?**																
No	94	52.5	21	53.8	45	60.0	5	27.8	10	71.4	4	50.0	6	42.9	3	27.3
Yes	85	47.5	18	46.2	30	40.0	13	72.2	4	28.6	4	50.0	8	57.1	8	72.7
**Have you experienced headaches?**																
No	99	55.3	19	48.7	43	57.3	8	44.4	11	78.6	1	12.5	8	57.1	9	81.8
Yes	80	44.7	20	51.3	32	42.7	10	55.6	3	21.4	7	87.5	6	42.9	2	18.2
**Have you experienced nausea?**																
No	157	87.7	32	82.1	65	86.7	15	83.3	14	100.0	7	87.5	13	92.9	11	100.0
Yes	22	12.3	7	17.9	10	13.3	3	16.7	0	0.0	1	12.5	1	7.1	0	0.0
**Have you experienced diarrhoea?**																
No	158	88.3	33	84.6	67	89.3	15	83.3	13	92.9	7	87.5	13	92.9	10	90.9
Yes	21	11.7	6	15.4	8	10.7	3	16.7	1	7.1	1	12.5	1	7.1	1	9.1
**Have you experienced insomnia?**																
No	108	60.3	25	64.1	38	50.7	12	66.7	11	78.6	3	37.5	10	71.4	9	81.8
Yes	71	39.7	14	35.9	37	49.3	6	33.3	3	21.4	5	62.5	4	28.6	2	18.2
**Have you experienced dizziness?**																
No	126	70.4	28	71.8	55	73.3	11	61.1	11	78.6	4	50.0	7	50.0	10	90.9
Yes	53	29.6	11	28.2	20	26.7	7	38.9	3	21.4	4	50.0	7	50.0	1	9.1
**Have you experienced nervousness?**																
No	130	72.6	29	74.4	53	70.7	12	66.7	12	85.7	6	75.0	10	71.4	8	72.7
Yes	49	27.4	10	25.6	22	29.3	6	33.3	2	14.3	2	25.0	4	28.6	3	27.3
**Have you experienced restlessness?**																
No	113	63.1	28	71.8	43	57.3	8	44.4	12	85.7	4	50.0	8	57.1	10	90.9
Yes	66	36.9	11	28.2	32	42.7	10	55.6	2	14.3	4	50.0	6	42.9	1	9.1
**Have you experienced depression?**																
No	150	83.8	31	79.5	66	88.0	13	72.2	13	92.9	6	75.0	13	92.9	8	72.7
Yes	29	16.2	8	20.5	9	12.0	5	27.8	1	7.1	2	25.0	1	7.1	3	27.3
**Have you experienced poor concentration?**																
No	141	78.8	29	74.4	58	77.3	11	61.1	13	92.9	8	100.0	13	92.9	9	81.8
Yes	38	21.2	10	25.6	17	22.7	7	38.9	1	7.1	0	0.0	1	7.1	2	18.2
**Have you experienced brain fog?**																
No	129	72.1	31	79.5	52	69.3	11	61.1	11	78.6	6	75.0	10	71.4	8	72.7
Yes	50	27.9	8	20.5	23	30.7	7	38.9	3	21.4	2	25.0	4	28.6	3	27.3
**Have you experienced rash on your skin?**																
Missing	1	0.6	1	2.6	0	0.0	0	0.0	0	0.0	0	0.0	0	0.0	0	0.0
No	134	74.9	25	64.1	57	76.0	13	72.2	11	78.6	8	100.0	11	78.6	9	81.8
Yes	44	24.6	13	33.3	18	24.0	5	27.8	3	21.4	0	0.0	3	21.4	2	18.2
**Have you experienced yellow eyes?**																
No	169	94.4	38	97.4	73	97.3	15	83.3	12	85.7	8	100.0	13	92.9	10	90.9
Yes	10	5.6	1	2.6	2	2.7	3	16.7	2	14.3	0	0.0	1	7.1	1	9.1

DTG, Dolutegravir.

The distribution of medication errors is presented in Table 3, disaggregated by province. Only 1.6% (*n* = 19) of respondents reported medication errors. Of those who reported medication errors, 42.1% (8/19) reported taking incorrect medication. Among those who took incorrect medication, 75% (6/8) reported experiencing problems, whereas one person was hospitalised 12.5% (1/8) and 25% (2/8) were contacted about the error.

**TABLE 3:** Medication errors reported by patients on the tenofovir disoproxil fumarate/lamivudine/dolutegravir regimen in the survey.

Variables	Overall		Gauteng		KwaZulu-Natal		Eastern Cape		Free State		Limpopo		Mpumalanga		North West	
	*n*	%	*n*	%	*n*	%	*n*	%	*n*	%	*n*	%	*n*	%	*n*	%
**Have you experienced any medication error while on the government chronic medication programme?**																
Missing	88	7.4	46	11.4	2	0.6	19	12.2	7	6.6	0	0.0	0	0.0	14	12.4
No	1085	91.0	351	87.3	316	97.8	135	89.5	97	91.5	28	100.0	63	98.4	95	84.1
Yes	19	1.6	5	1.2	5	1.5	2	1.3	2	1.9	0	0.0	1	1.6	4	3.5
**Did you take incorrect medication?**																
Missing	2	10.5	0	0.0	2	40.0	0	0.0	0	0.0	0	0.0	0	0.0	0	0.0
No	9	47.4	3	60.0	2	40.0	2	100.0	0	0.0	0	0.0	0	0.0	2	50.0
Yes	8	42.1	2	40.0	1	20.0	0	0.0	2	100.0	0	0.0	1	100.0	2	50.0
**If incorrect medication was taken, did you experience any problems?**																
No	2	25.0	1	50.0	0	0.0	0	0.0	1	50.0	0	0.0	0	0.0	0	0.0
Yes	6	75.0	1	50.0	1	100.0	0	0.0	1	50.0	0	0.0	1	100.0	2	100.0
**Were you ever hospitalised or treated due to the medic ation error?**																
No	7	87.5	2	100.0	1	100.0	0	0.0	1	50.0	0	0.0	1	100.0	2	100.0
Yes	1	12.5	0	0.0	0	0.0	0	0.0	1	50.0	0	0.0	0	0.0	0	0.0
**Did you receive the correct medication thereafter?**																
No	0	0.0	0	0.0	0	0.0	0	0.0	0	0.0	0	0.0	0	0.0	0	0.0
Yes	8	100.0	2	100.0	1	100.0	0	0.0	2	100.0	0	0.0	1	100.0	2	100.0
**Were you contacted about the medication error?**																
No	6	75.0	2	100.0	1	100.0	0	0.0	0	0.0	0	0.0	1	100.0	2	100.0
Yes	2	25.0	0	0.0	0	0.0	0	0.0	2	100.0	0	0.0	0	0.0	0	0.0

### Tenofovir disoproxil fumarate/emtricitabine/efavirenz regimen

A total of 1775 patients on the TEE regimen were interviewed (Table 4); the majority were women (75.4%, *n* = 1338). The highest proportion of the patients was in the age groups 30–39 (34%, *n* = 603) and 40–49 (31.4%, *n* = 558) years. Only 29.1% (*n* = 516) of the respondents reported being asked to change to the new antiretroviral regimen, whereas 65.7% (*n* = 1167) reported going for annual viral loads and 25.5% (*n* = 452) reported that they knew their viral load results. Among those who reported not going for their annual viral loads, the majority were from the Gauteng (41.8%, *n* = 191) and Eastern Cape (40.3%, *n* = 58) provinces.

**TABLE 4:** Participant characteristics of patients on the tenofovir disoproxil fumarate/emtricitabine/efavirenz regimen.

Variables	Overall		Gauteng		KwaZulu-Natal		Eastern Cape		Free State		Limpopo		Mpumalanga		North West	
	*n*	%	*n*	%	*n*	%	*n*	%	*n*	%	*n*	%	*n*	%	*n*	%
Number interviewed	1775		457		642		144		105		133		213		74	-
**Gender**																
Female	1338	75.4	355	77.7	434	67.6	127	88.2	92	87.6	110	82.7	150	70.4	64	86.5
Male	437	24.6	102	22.3	208	32.4	17	11.8	13	12.4	23	17.3	63	29.6	10	13.5
**Age group (years)**																
20–29	148	8.3	41	9.0	56	8.7	13	9.0	8	7.6	6	4.5	13	6.1	11	14.9
30–39	603	34.0	170	37.2	214	33.3	49	34.0	40	38.1	26	19.5	76	35.7	26	35.1
40–49	558	31.4	148	32.4	196	30.5	42	29.2	39	37.1	50	37.6	58	27.2	21	28.4
50–59	311	17.5	75	16.4	124	19.3	21	14.6	11	10.5	28	21.1	40	18.8	11	14.9
60–69	109	6.1	20	4.4	31	4.8	14	9.7	6	5.7	16	12.0	18	8.5	4	5.4
70–79	22	1.2	2	0.4	4	0.6	4	2.8	1	1.0	4	3.0	6	2.8	1	1.4
≥ 80	2	0.1	0	0.0	2	0.3	0	0.0	0	0.0	0	0.0	0	0.0	0	0.0
Missing	22	1.2	1	0.2	15	2.3	1	0.7	0	0.0	3	2.3	2	0.9	0	0.0
**Did your nurse ask if you would like to chang e to the new ARV medicine?**																
Missing	359	20.2	6	1.3	232	36.1	0	0.0	0	0.0	48	36.1	68	31.9	1	1.4
No	900	50.7	390	85.3	129	20.1	122	84.7	93	88.6	35	26.3	69	32.4	59	79.7
Yes	516	29.1	61	13.3	281	43.8	22	15.3	12	11.4	50	37.6	76	35.7	14	18.9
**Did you go for your annual bloods?**																
Missing	8	0.5	0	0.0	4	0.6	0	0.0	0	0.0	0	0.0	4	1.9	0	0.0
No	600	33.8	191	41.8	189	29.4	58	40.3	34	32.4	44	33.1	61	28.6	20	27.0
Yes	1167	65.7	266	58.2	449	69.9	86	59.7	71	67.6	89	66.9	148	69.5	54	73.0
**Do you know your viral load results?**																
Missing	43	2.4	0	0.0	30	4.7	0	0.0	0	0.0	4	3.0	8	3.8	0	0.0
No	1280	72.1	329	72.0	450	70.1	121	84.0	75	71.4	97	72.9	155	72.8	49	66.2
Yes	452	25.5	128	28.0	162	25.2	23	16.0	30	28.6	32	24.1	50	23.5	25	33.8

ARV, antiretroviral.

Table 5 presents the distribution of medication errors, stratified by province. The proportion of respondents reporting medication errors while on the TEE regimen was low (1.2%, *n* = 22), with Gauteng reporting the highest proportion (2.6%, 12/457). Of these, 36.4% (*n* = 8) reported taking incorrect medication, with 87.5% (7/8) of these reporting drug-related side effects. The number of patients reporting hospitalisation or treatment due to medication error (25%, 2/8), receiving the correct medication thereafter (75%, 6/8), and being contacted by the service provider about the medication error (37.5%, 3/8), was low.

**TABLE 5:** Medication errors reported by patients on the tenofovir disoproxil fumarate/emtricitabine/efavirenz regimen in the survey.

Variables	Overall		Gauteng		KwaZulu-Natal		Eastern Cape		Free State		Limpopo		Mpumalanga		North West	
	n	%	n	%	n	%	n	%	n	%	n	%	n	%	n	%
**Have you experienced any medication error while on the government chronic medication pro gramme?**																
Missing	34	1.9	12	2.6	11	1.7	3	2.1	0	0.0	2	1.5	4	1.9	2	2.7
No	1719	96.8	433	94.7	625	97.4	140	97.2	104	99.0	131	98.5	208	97.7	71	95.9
Yes	22	1.2	12	2.6	6	0.9	1	0.7	1	1.0	0	0.0	1	0.5	1	1.4
**Did you take the incorrect medication?**																
Missing	3	13.6	3	25.0	0	0.0	0	0.0	0	0.0	0	0.0	0	0.0	0	0.0
No	11	50.0	6	50.0	3	50.0	1	100.0	0	0.0	0	0.0	1	100.0	0	0.0
Yes	8	36.4	3	25.0	3	50.0	0	0.0	1	100.0	0	0.0	0	0.0	1	100.0
**If incorrect medication was taken, did you experience any drug side effects?**																
No	1	12.5	0	0.0	1	33.3	0	0.0	0	0.0	0	0.0	0	0.0	0	0.0
Yes	7	87.5	3	100.0	2	66.7	0	0.0	1	100.0	0	0.0	0	0.0	1	100.0
**Were you ever hospitalised or treated due to the medication error?**																
No	6	75.0	2	66.7	3	100.0	0	0.0	1	100.0	0	0.0	0	0.0	0	0.0
Yes	2	25.0	1	33.3	0	0.0	0	0.0	0	0.0	0	0.0	0	0.0	1	100.0
**Did you receive the correct medication thereafter?**																
No	2	25.0	0	0.0	1	33.3	0	0.0	1	100.0	0	0.0	0	0.0	0	0.0
Yes	6	75.0	3	100.0	2	66.7	0	0.0	0	0.0	0	0.0	0	0.0	1	100.0
**Did the service provider contact you about the medication error?**																
No	5	62.5	1	33.3	3	100.0	0	0.0	1	100.0	0	0.0	0	0.0	0	0.0
Yes	3	37.5	2	66.7	0	0.0	0	0.0	0	0.0	0	0.0	0	0.0	1	100.0

## Discussion

This survey describes ADRs, and medication errors reported by HIV-infected patients on ART in the CCMDD programme in South Africa. Though some work exists on medication errors,^13,14^ quantifiable data on medication errors from large HIV programmes from low- and middle-income countries are sparse. The number of ADRs and medication errors reported by patients was low, most probably because the CCMDD programme enrols HIV patients who are stable on their treatment. By design, ADRs were largely reported in detail by patients on the TLD regimen, as it is a newer regimen and rollout is ongoing. The CCMDD programme provides a mechanism to support health systems with minimal errors and facilitating fewer patients needing to visit healthcare facilities, particularly in this coronavirus disease 2019 (COVID-19) pandemic period. At the start of the TLD regimen rollout, DTG-containing regimens had been thought to increase the risk of neural tube defects in the offspring of female users and therefore clinicians and prescribers were advised to counsel all women of childbearing potential on these risks.^15,16^ Despite this, only 55.4% of females in this survey who were in the childbearing potential age category reported receiving this risk-associated counselling. The DTG guidelines have since been updated to show no significant difference in neural tube defects between DTG and non-DTG regimens.^17^

Although low in numbers, weight gain, headaches, insomnia, restlessness, dizziness, brain fog, nervousness, skin rash and poor concentration were commonly reported among those on the TLD regimen. Our findings are in harmony with prior research that shows neuropsychiatric ADRs in patients taking DTG-containing regimens.^18,19^ Adverse drug reactions in the CCMDD programme could be low due to the caution observed in enrolling only stable patients on ART. Additionally, when any ADRs are experienced, the patients are immediately transferred to the standard primary health care clinics to be managed by clinicians until they stabilise again.

Medication errors reported under both the TLD and TEE regimens in the CCMDD programme were low. Though there are limited data on medication errors reported from low- and middle-income countries’ HIV programmes, a study in Australia examined medication errors in general in a large public hospital that implemented a system to monitor and reduce their occurrence.^13^ A previous study from the Netherlands observed a high number of medication administration errors in nursing homes.^20^ In a literature review on the management and care of hospitalised HIV-infected patients receiving ART in high-income countries, a large number of medication errors was reported.^21^ It may be that medication errors reported in the CCMDD programme are low due to close supervision and adherence to the standard operating procedures for dispensed medication.

This evaluation of ADRs and medication errors from the CCMDD programme has several limitations. Due to the COVID-19 pandemic, the consenting and interviewing process was telephonic and response bias may have occurred due to fatigue associated with non-face-to-face (telephonic) platforms. While the survey was designed to collect data as objectively as possible, it is likely that social desirability bias (patients responding favourably to questions) occurred. The risk of ADRs and medication errors is highest at the start of a new treatment regimen. Since only stable chronic disease patients are recruited in the CCMDD programme, high-risk patients who are likely to have poor outcomes are under-represented in this cohort. Additionally, those encountering challenges within the system or the medication were more likely to respond. The majority of the participants in this survey had been on the TEE regimen for longer than those on TLD, which has only been available for a few years. The sampling frame of participants for the survey was generated from the CCMDD database during the COVID-19 pandemic. Hence, bias due to the pandemic, such as the national lockdown and staff absenteeism, may have been missed. Nevertheless, the findings are encouraging and highlight the purpose of the CCMDD programme, to ensure widespread access to ART and other chronic medication for patients accessing public healthcare.^22^ The differentiated service delivery model is commonly used in the management and care of PLWH who require ART. In brief, the differentiated service delivery model seeks to improve service delivery by offering patient-centred care through optimised drugs and care delivery.^23^ The CCMDD programme resembles the differentiated service delivery model, but has expanded successfully to include a broader spectrum of chronic diseases beyond HIV. Patient-centred care has been shown to significantly improve health outcomes in Mozambique, the Democratic Republic of Congo, Malawi and South Africa.^24^ In the United Kingdom, a study found that patients in nursing homes who received diabetic medication rarely reported medication errors.^25^ In Australia, implementation of multiple patient-centred and system redesign strategies significantly reduced medication errors across the health service.^13^ This suggests that the implementation of the CCMDD programme in South Africa can be expected to become more critical as it expands its service offerings.

## Conclusion

Findings from this evaluation highlight the relatively low frequency of ADRs and medication errors among stable HIV patients on treatment in the CCMDD programme. The CCMDD programme strives to ensure that ADRs and medication errors are minimised, as they focus on programme expansion to include other diseases. This will further alleviate in-facility congestion and improve alternate access to treatment and retention in care, through decentralised medication service delivery, especially now during the COVID-19 pandemic.

Knowing the frequency of ADRs and medication errors is critical in enhancing the CCMDD programme through continuous evaluation and enhancement of quality control measures for patient safety. Although the CCMDD programme has reduced congestion at facilities and freed up time for clinicians to focus on other healthcare services, there is a need to continuously monitor the timeframes for reporting ADRs and medication errors. This will allow for prompt responses by clinicians and minimise the impact of ADRs and medication errors on patients.
